# Role of *PAX7* Gene rs766325 and rs4920520 Polymorphisms in the Etiology of Non-syndromic Cleft Lip and Palate: A Genetic Study

**DOI:** 10.1055/s-0042-1748531

**Published:** 2022-07-15

**Authors:** Mahamad Irfanulla Khan, Prashanth CS, Narasimhamurty Srinath

**Affiliations:** 1 Department of Orthodontics and Dentofacial Orthopedics, The Oxford Dental College, Bengaluru, India; 2 Department of Orthodontics and Dentofacial Orthopedics, DAPM R. V. Dental College, Bengaluru, India; 3 Department of Oral and Maxillofacial Surgery, Krishnadevaraya College of Dental Sciences, Bengaluru, India

**Keywords:** non-syndromic, cleft lip and palate, *PAX7*, DNA extraction, genotyping

## Abstract

Non-syndromic cleft lip and palate (NSCLP) is one of the most common birth defects in humans with an overall prevalence of ∼1 in 700 live births around the world. The etiology of NSCLP is complex involving multiple genes, environmental factors, and gene-to-gene interactions. Several genome-wide associations (GWA) studies have shown the association of the paired box 7 ( *PAX7* ) gene in the etiology of cleft lip and palate in different populations worldwide. However, there are no reported studies on the association between the rs766325 and rs4920520 polymorphisms and the risk of developing NSCLP in the Indian population. Hence, the present study aimed to test for the probable association between rs766325 and rs4920520 polymorphisms among NSCLP Indian population using a case-parent trio design. Forty case-parent trios were selected from the cleft lip and palate center based on the inclusion and exclusion criteria. Genomic DNA was isolated from the cases and their parents. The rs766325 and rs4920520 polymorphisms of the *PAX7* gene were analyzed for their association using the MassARRAY analysis. The statistical analysis was done using the PLINK software. The rs766325 and rs4920520 polymorphisms were tested for the Hardy–Weinberg equilibrium. None of the polymorphisms showed any statistical significance. Hence, the rs766325 and rs4920520 polymorphisms of the *PAX7* gene were found to be not associated with NSCLP in the Indian case-parent trios.

## Introduction

Non-syndromic cleft lip and palate (NSCLP) is one of the most common congenital anomalies of the human face with an overall prevalence of ∼1 in 700 live births worldwide. 1 These clefts not only result in facial deformities but also occur with complications, such as difficulty in feeding, speech, and esthetic problems. 2 3 Overall, 70% of the clefts cases are non-syndromic, whereas 30% are syndromic. 4 5

The prevalence of NSCLP varies among geographic regions and socioeconomic status. 6 Native American Indians, Japanese have a relatively higher prevalence, Europeans with intermediate, and the lowest in Africans. 7 The incidence of clefts in India is around 1:800 to 1:1000. 8 The etiology of NSCLP is complex involving several genes and environmental factors. 9 10 In India, consanguinity is an important etiological factor. Neela et al 11 reported in a 13-year retrospective study from a cleft center that 20.02% of cleft patients had consanguineous parents.

In humans, the paired box 7 ( *PAX7* ) gene affects the development of the neural crest development in the craniofacial region. 12 Animal studies also demonstrated the deficiency of *PAX7* resulting in the defect in the formation of the nasal cavity, lacrimal bones, and maxilla. 13

High-risk polymorphisms rs766325 and rs4920520 of the *PAX7* gene are involved in the etiology of NSCLP and have been evaluated in different populations worldwide. The present literature review reveals there are no reported studies on the association between these polymorphisms and the risk of developing NSCLP in the Indian population. Hence, we selected these high-risk polymorphisms from the literature (previous studies) to test for the probable association between rs766325 and rs4920520 polymorphisms of the *PAX7* gene with NSCLP in the Indian population using a case-parent trio design.

## Materials and Methods

### Sample Description

Forty case-parent trios were selected from the cleft lip and palate clinic of the Mallige Medical Centre (Bengaluru, India). Clinicians and geneticists examined each case to exclude the syndromic forms of clefts. The present study was approved by the Institutional Review Board (IRB No. 230/Vol-2/2017). The research followed the world medical association Declaration of Helsinki on medical experiments and ethics. The etiology of orofacial clefts is studied generally irrespective of the phenotypic features, cleft lip (unilateral or bilateral), cleft lip with cleft palate, and very few studies only on cleft palate. In the present study, the phenotypic features included were cleft lip and palate (CLP), cleft lip (CL), and cleft palate (CP) only.

Patients with cleft lip and palate (samples: 21), cleft lip (samples: 13), and cleft palate only (samples: 6) were included in this study. Patients with a congenital anomaly and any syndromes associated with the cleft were excluded. Written informed consent for blood withdrawal was obtained from all cases and their parents (trios).

### Genotyping Analysis

The rs766325 and rs4920520 polymorphisms ( Table 1 ) were selected from the NCBI database ( www.ncbi.nlh.nih.gov/snp/ ) and previous studies. The genomic DNA was extracted from 3 mL of peripheral blood using the QIAamp DNA Mini Kit (Qiagen Inc, CA, USA) following the manufactures instructions. The polymorphisms rs766325 and rs4920520 were genotyped using the Agena Bio MassARRAY (Agena Bioscience, Inc., San Diego, CA, USA) platform using matrix-assisted laser desorption/ionization-time of flight (MALDI-TOF) mass spectrometry.

**Table 1:** List of the polymorphisms tested

Gene	Polymorphisms	Position	Alleles	Ancestral allele	MAF
*PAX7*	rs766325	1:18629964	G/A	A	0.49
*PAX7*	rs4920520	1:18603348	G/A	G	0.39

Abbreviations: A, adenine; G, guanine; MAF, minor allele frequency; *PAX7* , paired box 7.

### Statistical Methods

Forty case-parent trios underwent the Hardy–Weinberg equilibrium (HWE) analysis and minor allele frequency (MAF) determination. The HWE, MAF, allelic transmission disequilibrium test (TDT), and parent-of-origin effects in all case-parent trios, were calculated using the PLINK software (version 1.07). 14 The 95% confidence interval (95%CI) for estimated odds ratios (ORs) was calculated, and a *P* -value < 0.05 was considered to be statistically significant.

## Results

The rs766325 and rs4920520 were tested for genotyping and followed the HWE test ( Table 2 ). Parent-of-origin effects were investigated by stratifying the information of transmitted (T) and untransmitted (UT) alleles by parental source for these two polymorphisms. The family-based TDT results ( Table 3 ) revealed no significant association of rs766325 and rs4920520 with NSCLP in the Indian case-parent trios.

**Table 2:** Hardy–Weinberg equilibrium test of the polymorphisms analyzed

Gene	Polymorphisms	A1	A2	Genotypes	O (HET)	E (HET)	HWE *p* -Value
*PAX7*	rs766325	A	G	UNAFF: 14/33/33 AFF: 6/16/18	0.4125	0.4718	0.2468
*PAX7*	rs4920520	A	G	UNAFF: 16/33/31 AFF: 7/17/16	0.4125	0.4824	0.2449

Abbreviations: A, adenine; A1, major allele (wild allele); A2, minor allele (mutant); AFF, affected; E (HET), Expected heterozygosity; G, guanine; HWE, Hardy–Weinberg equilibrium; O (HET), observed heterozygosity; UNAFF, unaffected.

**Table 3:** Association between the rs766325 and rs4920520 of *PAX7* gene and NSCLP

CHR	Polymorphisms	A1	A2	T	U	OR (95% CI)	CHISQ	*p* -Value
1	rs766325	A	G	19	10	1.9	2.793	0.09467
1	rs4920520	A	G	18	13	1.385	0.8065	0.3692

Abbreviations: A, adenine; A1, major allele (wild allele); A2, minor allele (mutant); CHISQ, Chi-square; CHR, chromosome number; CI, confidence interval; G, guanine; NSCLP, non-syndromic Cleft lip and palate; OR, odds ratio; T, minor allele transmitted; U, minor allele un-transmitted.

Note: *p* -Value < 0.05 is significant.

## Discussion

The rs766325 and rs4920520 of the *PAX7* gene were previously identified to be associated with the risk of NSCLP in different populations. In the current study, we used 40 NSCLP trios to test for the probable association between rs766325 and rs4920520 polymorphisms of the *PAX7* gene among NSCLP Indian children using a case-parent trio design.

The family studies for the Indian population reported that several genes such as *CRISPLD2, RUNX2, SOX1-OT, MAPK4, CTIF, MYO5B, SMAD7, LOXHD1* , and *SKA1* are not associated with NSCLP in Indian multiplex families. 15 16 17 However, there are no reported case-parent trio studies of the *PAX7* gene in the Indian population. Case–parent trio design studies are generally used in genetics, in which the affected children and their parents (father and mother) are genotyped. The case–parent trio study design offers the advantage of testing maternal versus paternal effects, parent of origin effects, and it minimizes issues of confounding that plague traditional case–control designs. 18

Several studies reported the *PAX* genes are involved in craniofacial morphogenesis through cellular proliferation, migration, and the regulation of differentiation programs during embryonic development. The paired box 7 ( *PAX7* ) gene belong to the *PAX* gene family and plays a critical role during fetal development as well as in neural crest development. So, any embryological disturbance in the neural crest development may lead to the development of the oral clefts such as cleft lip and cleft palate.

The *PAX7* gene along with *PAX3* plays an essential role during craniofacial development in the regulation of morphogenesis, survival, patterning, and specification of the frontonasal structures. 19 Transcription factors of these *PAX7* and *PAX3* help to maintain the proliferative cells during the development in embryonic and fetal muscles of the trunk and limbs. 20

Genome-wide association studies (GWAS) have successfully provided evidence for the genetic etiology of NSCLP. A meta-analysis showed single nucleotide polymorphisms (SNPs) yielding the greatest significance in specific regions in and around the *PAX7* gene. Considering the gene–gene interactions (G × G), the results showed nominally significant in the Asian group the *MAFB* gene SNP rs17820943 interacted with the *PAX7* gene SNP rs4920520. However, the results in the cleft palate group were not significant. 21 In the European case–parent trios, when they tested for G × G interaction, the polymorphisms of the *PAX7* showed no significant association with NSCLP. 22

The *PAX7* gene has been associated with cleft lip and palate in some association studies, 23 but not others. A possible reason for these conflicting results may be due to the sample size, ethnicity, design of the test, and classification of non-syndromic oral clefts.

Hence, the rs766325 and rs4920520 of the *PAX7* gene were tested in the NSCLP patients using a case–parent trio design as there are no reported trio studies on the role of these polymorphisms in the Indian population. The present study suggests that rs766325 and rs4920520 of PAX7 are not associated with NSCLP in the Indian population.

Several studies reported no significant association of rs766325 and rs4920520 of *PAX7* with NSCLP in different populations worldwide. A study by Sull et al 24 showed that the rs766325 was significantly associated with non-syndromic cleft lip and palate case–parent trios of the Taiwanese, Singaporean, Korean, and Maryland population. In a case–control study by Guoet al 25 with 602 individuals with non-syndromic orofacial clefts and 510 controls from northern China, the rs766325 showed no significance.

Duan et al 26 in their study consisted of 144 non-syndromic cleft palate only (NSCPO) trios from the Western Han Chinese population reported no association of rs766325 with NSCPO. In a case–parent trio study by Beaty et al, 23 297 European and Asian trios showed that the rs766325 was not associated with non-syndromic oral clefts. Butali et al 27 recruited individuals from Iowa, Japan, Mongolia, and the Philippines found there was no significant association of rs766325 with NSCLP.

In a northern Chinese population of non-syndromic orofacial clefts, 25 and also in a study by Butali et al, the rs4920520 showed no significant association. 27

The present study results showed that the rs766325 and rs4920520 of the *PAX7* gene are not associated with the risk of NSCLP among the Indian population, similar to the several previous genetic studies. However, the limitations of our study are a relatively smaller sample size and the analysis of only two high-risk polymorphisms. Further studies should be conducted with more polymorphisms of the *PAX7* gene with a larger sample size for the Indian population to confirm the role of the *PAX7* gene in the etiology of NSCLP.

## Conclusion

In the present study, the rs766325 and rs4920520 polymorphisms of the *PAX7* gene were found to be not associated with NSCLP among the Indian case–parent trios. Further work will be required to confirm the role of these polymorphisms with a bigger sample size.
